# Climate, conflict and displacement in the Sahel 

**DOI:** 10.2471/BLT.24.292700

**Published:** 2025-04-01

**Authors:** Ejemai Eboreime, Omolayo Anjorin, Chisom Obi-Jeff, Tunde M Ojo, Attila Hertelendy

**Affiliations:** a Department of Psychiatry, Faculty of Medicine, Dalhousie University, 5909 Veterans’ Memorial Lane, 8th Floor Abbie J. Lane Memorial Building, B3H 2E2 Halifax, NS, Canada.; b Institute of Psychiatry, Psychology and Neuroscience, King’s College London, London, England.; c Brooks Insights, Abuja, Nigeria.; d Department of Public Health, Federal Ministry of Health and Social Welfare, Abuja, Nigeria.; e Department of Information Systems and Business Analytics, Florida International University, Miami, United States of America.

The Sahel, a semi-arid belt stretching from Senegal to the Sudan, is one of the world’s most climate-vulnerable areas.^1^ Rising temperatures, desertification and ecosystem losses, combined with social, economic and political factors, are fuelling violence and forced displacement, with severe consequences for health and well-being.^1^ Despite contributing minimally to global carbon emissions, Sahelian communities bear a disproportionate burden of climate change, compounding long-standing humanitarian challenges linked to armed conflict and violent extremism.^1^ Understanding these dynamics is essential for developing effective interventions, as the area’s instability has far-reaching implications for global migration, security and public health.^2^

Environmental shifts due to climate change have intensified competition between farmers and herders over scarce resources, leading to deadly clashes in countries like Burkina Faso, Mali and Nigeria.^3^ Furthermore, extremist groups have taken advantage of climate-related grievances and resource scarcity to expand their influence, further contributing to instability.^4^ The resulting violence has devastated local communities,^2^ with millions of individuals forced into multiple displacement^5^ as climate impacts and conflict make it increasingly difficult to sustain livelihoods and ensure safety. Without intervention, climate models suggest that sub-Saharan Africa could see up to 85 million people displaced due to environmental factors by 2050, underlining the need for proactive resilience and adaptation measures.^6^

Displaced communities experience increased vulnerability to malnutrition, waterborne diseases and mental health challenges, with limited access to basic health-care services.^2^ The rapid influx of displaced populations into some cities has placed enormous strain on already overstretched urban infrastructure and services,^1^ leading to increased health risks, food and water insecurity and social tensions.^7^ Additionally, desertification and deforestation accelerate biodiversity loss, reduce agricultural productivity, increase the likelihood of diseases^1^ and worsen health conditions.^8^ These impacts threaten progress on key development goals related to health, equality and sustainable communities, trapping affected populations in a vicious cycle of vulnerability and displacement.^1^

Addressing the interconnected challenges of climate change, conflict and displacement in the Sahel requires sustainable, evidence-based solutions. A planetary health approach considers both environmental and human health impacts, offering a holistic way to reduce vulnerability and strengthen resilience.^8^ Climate adaptation strategies must align with the region’s environmental and socioeconomic realities to effectively support the most vulnerable populations.^1^ Interventions should provide immediate relief while fostering long-term stability and resilience against future climate and security threats. A coordinated, multisectoral and multilevel approach implemented by governments with support from regional bodies and global partners that integrates climate adaptation with conflict prevention and social protection programmes is vital to creating sustainable pathways for affected communities.

Existing frameworks provide structured strategies to mitigate climate-related challenges in the Sahel. The *African Union climate change and resilient development strategy and action plan (2022–2032)*^9^ promotes climate-resilient agriculture, early warning systems and sustainable land management to combat desertification and improving food security, addressing both environmental and socioeconomic drivers of displacement. Similarly, water management strategies under the Niger Basin Authority have demonstrated the value of transboundary cooperation through initiatives that improve water access and irrigation systems across member countries.^10^ These efforts align with the African Union’s strategy, which promotes climate-resilient agriculture and sustainable water management practices to support vulnerable farming communities and reduce displacement pressures.^9^

Urban adaptation policies are key to enhancing infrastructure and service delivery in rapidly growing cities, ensuring displaced populations have access to essential services, employment and secure housing.^8^ Given the scale of displacement in the Sahel, establishing long-term resettlement and integration strategies is essential. While some individuals may eventually return to their original homes, for many, permanent relocation will be necessary due to environmental degradation and ongoing instability. Planned urban integration is a cornerstone of durable solutions. The Global Compact on Refugees emphasizes the importance of inclusive policies that support long-term integration while upholding the rights of displaced individuals.^11^
